# The Impact of Undernutrition and Anemia on HIV-Related Mortality Among Children on ART in Sub-Saharan Africa: A Systematic Review and Meta-Analysis

**DOI:** 10.1007/s44197-024-00321-6

**Published:** 2024-11-14

**Authors:** Sisay Moges, Bereket Aberham Lajore, Abera Feyisa Oleba, Abraham Samuel Godebo, Mengistu Lodebo Funga

**Affiliations:** 1 Department of Family Health, Hossana College of Science, Hosanna, Ethiopia; 2 Department of Clinical Nursing, Hossana College of Science, Hosanna, Ethiopia; 3 Department of Emergency and Critical Care, Hossana College of Science, Hosanna, Ethiopia; 4 Department of Midwifery, Hossana College of Science, Hosanna, Ethiopia

**Keywords:** Effect of undernutrition, Effect of anemia, Risk factor for HIV mortality, Children, Sub-saharan Africa

## Abstract

**Background:**

Although there have been significant advancements in providing HIV-infected children with access to antiretroviral therapy (ART), the mortality rates have remained unacceptably high. Inadequate nutrient intake or absorption is a widespread problem in several African nations, resulting in undernutrition and anemia. However, the pooled effect of malnutrition and anemia on HIV-related death related to children receiving ART was not investigated in sub-Saharan Africa.

**Methods:**

We searched multiple electronic databases (PubMed/MEDLINE, Embase, CINAHL, and Web of Science) for observational studies published between January 1, 2010, and April 24, 2024 that reported the risk factors or effects of undernutrition and, anemia on HIV-related mortality among children. Study selection, data extraction, and quality evaluation were carried out separately by two reviewers. A meta-analysis was conducted using random effect models.

**Results:**

The review included 27 studies with a combined total of 61,796 study participants. The study findings showed that severe wasting (HR: 2.49; 95% CI: 1.87–3.30), being underweight (HR: 2.11; 95% CI: 1.64–2.72), and Anemia (HR: 2.58; 95% CI: 2.08–3.19) were highly linked to HIV-related death among children. The risk of death due to anemia was greater among children under the age of 5 years than older children.

**Conclusion:**

Undernutrition and anemia in sub-Saharan African children increased the risk of HIV-related death. The impact of malnutrition and anemia among under 5 years old children with HIV/AIDS was more pronounced, suggesting that these conditions at this early age can have more serious consequences for a child’s survival. The importance of combining nutrition with HIV treatment programs in sub-Saharan African countries is crucial.

## Background

The HIV/AIDS epidemic continues to be a significant public health challenge in sub-Saharan Africa, with children being particularly vulnerable to its impacts [1]. Although there has been significant advancement in providing HIV-infected children in this area with access to antiretroviral therapy (ART), the mortality rates have remained unacceptably high [2]. According to the World Health Organization (WHO), in the African region, an estimated 1.3 million children aged 0–14 were living with HIV at the end of 2022, and approximately 69,000 children died of AIDS-related illnesses [3]; a mortality rate of 7.9% was reported in sub-Saharan African countries [4]. Although ART has greatly increased the survival rates and quality of life of HIV-infected individuals, its effectiveness in children is frequently impacted by factors such as undernutrition and anemia [5].

Inadequate nutrient intake or absorption is a widespread problem in several African nations, resulting in undernutrition and anemia [6]. Undernutrition in the presence of HIV can worsen the disease, weaken the immune system, and decrease the effectiveness of antiretroviral medications [7]. The complex interplay between HIV and undernutrition forms a harmful cycle, with each condition worsening the effects of the other, possibly resulting in higher rates of illness and death [8, 9]. However, malnutrition and anemia are not the sole causes of HIV-related mortality among children but serve as critical markers in a complex interplay of factors. They indicate underlying vulnerabilities, such as weakened immune systems and poor overall health, which make children more susceptible to opportunistic infections and hinder their response to antiretroviral therapy (ART) [10–12]. This interplay involves co-existing infections, delayed ART initiation, and inadequate healthcare access, all contributing to higher mortality risks [13, 14]. While malnutrition and anemia reflect these broader issues, they are part of a multifaceted web of clinical and socio-economic factors driving poor health outcomes in HIV-infected children. Anemia, a frequent coexisting condition in children with HIV, is defined by a lower amount of hemoglobin in the bloodstream, which can lead to tiredness, lack of strength, and decreased cognitive abilities [15, 16]. Anemia in HIV-infected children has diverse causes, including decreased red blood cell production, increased rates of blood cell destruction, and a lack of nutrients [17, 18].

Previous studies in Africa have inconsistent across different settings. For example studies, such as [13, 19, 20], reported a non-significant association between malnutrition and HIV-related mortality among children, while others found a significant link, highlighting the variability in outcomes across different settings [21–23]. Similarly, for anemia, certain studies [24, 25] observed no significant association with mortality, whereas others demonstrated a strong correlation between anemia and increased risk of death in children with HIV [21, 22]. These mixed findings emphasize the need to interpret the results within the specific contexts and populations studied. Nevertheless, it is uncertain how applicable these results are to the wider sub-Saharan African setting and how they specifically relate to children receiving ART. This issue becomes more complex due to the socioeconomic conditions in sub-Saharan Africa. Poverty, lack of food, and restricted healthcare access are common issues that can worsen undernourishment and anemia and make managing HIV difficult [26, 27]. Therefore, this review.provide valuable insights into the role of malnutrition and anemia in HIV-related mortality among children, highlighting key knowledge gaps and guiding evidence-based interventions and policies. The findings have the potential to inform clinical practice, support program development, and shape policy creation in this critical area of child health.

## Methods and Materials

### Eligibility Criteria

Studies were included based on predefined criteria regarding population, comparators, outcomes, study design, settings, period, and language. The target population comprised HIV-infected children (aged 0–15 years) receiving ART, and studies must have included participants from one or more countries in sub-Saharan Africa. Undernutrition, including wasting and underweight, as well as anemia (hemoglobin < 10 g/dL), were considered key exposure factors. The primary outcome was all-cause mortality, used to assess and compare the risk of death associated with these specific exposures. The eligible studies included retrospective cohort, case-control, and cross-sectional studies. Moreover, theses and dissertations were also included. Studies involving both study participants with TB and HIV infection simultaneously, single case reports, case series, reviews, commentaries, editorials, studies not reporting the association between malnutriton and/or anemia with HIV related mortality, and studies with insufficient data to calculate effect estimates were excluded. Studies carried out in countries in sub-Saharan Africa after 2010 until now and released in the English language were included. Furthermore, any studies that could not be accessed after two attempts at communication with the main author or corresponding author were also not included in the review. The inclusion and exclusion criteria aimed to pinpoint important and high-quality research focused on risk factors in pediatric patients with HIV infection.

### Search Strategy and Information Sources

We searched multiple electronic databases such as PubMed/MEDLINE, Hinari CINAHL, Embase, Google Scholar, African Journals Online (AJOL), OpenGray and Science Direct databases for peer-reviewed articles published from January 1, 2010, to April 24, 2024. We selected the period from January 2010 to April 24, 2024, because 2010 marks the introduction of more robust and effective ART combinations by the WHO, along with a significant shift in treatment strategies. Furthermore, prospective articles of interest were manually searched through the reference lists of eligible studies. Two authors (SM and BAL) independently searched. The search strategy was developed in consultation with a medical librarian and will include a combination of Medical Subject Headings (MeSH) terms and free-text keywords. The search terms were grouped into four main categories: 1) population: HIV-infected children; 2) exposure: undernutrition and anemia; 3) outcome: mortality; and 4) setting: sub-Saharan Africa. The following keywords were used: HIV/AIDS; death OR survival; predictor OR associated factor OR risk factors; OR determinants; effect of undernutrition; effect of anemia; antiretroviral therapy OR ART; pediatrics OR children; under five years OR 15 years [African countries]. Methodological terms such as prospective, retrospective, cohort, and cross-sectional studies were also included. Using Boolean operators (AND, OR), these keywords were combined to generate extensive search strings that will find pertinent research in a variety of databases. For example, the following search strategy was employed in the PubMed database: (HIV[MeSH] OR HIV Infections [MeSH] OR HIV [tiab] OR Human Immunodeficiency Virus[tiab] OR AIDS [tiab]) AND (Child[MeSH] OR Pediatrics[MeSH] OR Child * [tiab] OR Pediatric* [tiab] OR Pediatrics* [tiab]) AND (Malnutrition[MeSH] OR Nutritional Status [MeSH] OR Anemia [MeSH] OR Malnutrition [tiab] OR Undernutrition[tiab] OR Nutritional Deficiency* [tiab] OR Anemia [tiab] OR Anemia[tiab]) AND (Mortality[MeSH] OR Death[MeSH] OR Mortality* [tiab] OR Death* [tiab] OR Survival [tiab]) AND (Antiretroviral Therapy, Highly Active [MeSH] OR Anti-Retroviral Agents[MeSH] OR Antiretroviral Therapy* [tiab] OR ART [tiab] OR HAART[tiab]) AND (Africa South of the Sahara” [MeSH] OR Sub-Saharan Africa* [tiab] OR names of individual sub-Saharan African countries). The retrieved studies were imported and managed using EndNote XX reference management software. The search was conducted between January 2024 and April 2024 to identify potentially eligible studies for the systematic review and meta-analysis. This search strategy will be adapted for use in other databases, taking into account their specific indexing systems and search functionalities.

### Screen and Selection Process

The screening and selection of studies followed systematic and rigorous approaches. Two reviewers (SM and BAL) independently reviewed the titles and abstracts of all retrieved records from the databases and manually searched them against the predefined eligibility criteria. All identified articles were entered into the EndNote XX database, and any duplicated entries were eliminated. Documentation of the reasons for exclusion was completed at this point. Any discrepancies or disagreements in selection between the two reviewers were resolved through discussion and consensus.

### Risk of Bias and Quality Assessment

The risk of bias and quality of the studies included in our review were meticulously evaluated by two independent reviewers utilizing the Newcastle‒Ottawa Scale (NOS) [21] for observational studies. The NOS assesses the risk of bias in three domains, including the selection of the study groups, the comparability of the groups, and the ascertainment of the outcome of interest. The NOS framework allows for a comprehensive assessment, allocating up to four stars for ‘Selection,’ two for ‘Comparability,’ and three for ‘Outcome’ categories. The results are presented in the ‘Summary of Findings’ table.

### Data Extraction

A Microsoft Excel spreadsheet was used for data extraction. Two authors (SM and BAL) independently extracted data from the included studies using a predefined checklist. The following data were extracted from each primary study: first author’s name, publication year, study country, and sample size. To ensure consistency in the data extraction process, both reviewers independently extracted data from the first three eligible articles. Any discrepancies or disagreements in selection between the two reviewers were resolved through discussion and consensus. Subsequently, they met to compare results, clarify any discrepancies, and refine the abstraction form if necessary. After this initial calibration, one reviewer extracted data from the remaining articles, and the second reviewer verified the extracted information.

### Outcome Variables and Effect Measures

This research focused on the pooled impact of severely wasting, underweight and anemia on HIV related mortality, as documented in primary studies among children on ART in sub-Saharan African nations. Severe wasting is defined as a weight for height (WHZ) measurement of < -3 SD, underweight is defined as a weight for age (WHZ) measurement of < -2 SD [28], and anemia is defined as a hemoglobin level of less than 10 g/dl [29]. The hazard ratio (HR) was a suitable measure of effect for our study. A random-effects model was used to combine the mortality risk for each selected exposure variable from various studies, taking into account potential heterogeneity between the studies.

### Data Synthesis and Analysis

A narrative synthesis summarized the study characteristics, populations, study countries, outcome assessments, and key findings. A meta-analysis was conducted using Stata software version 17 to estimate the role of undernutrition and anemia on HIV-related mortality. The hazard ratios (HRs) with 95% confidence intervals (CIs) were computed. Hazard ratio (HRs) from primary studies were log-transformed (logHR) to obtain the effect sizes and corresponding standard errors. Forest plots visually display the pooled estimates. Heterogeneity was assessed using Cochran’s Q statistic, the I2 statistic, and the chi-square test (*P* < 0.05). Heterogeneity levels were classified as low (I^2^: 0–25%), moderate (I^2^: 25–50%), or high (I^2^: ≥50%) [30, 31]. The DerSimonian‒Laird method was used to estimate variance in random effects models. Subgroup analyses explored potential sources of heterogeneity, considering study countries and study participant age. The report is presented in the text, tables and figures.

## Results and Discussion

### Selection of Studies

**Fig. 1:**
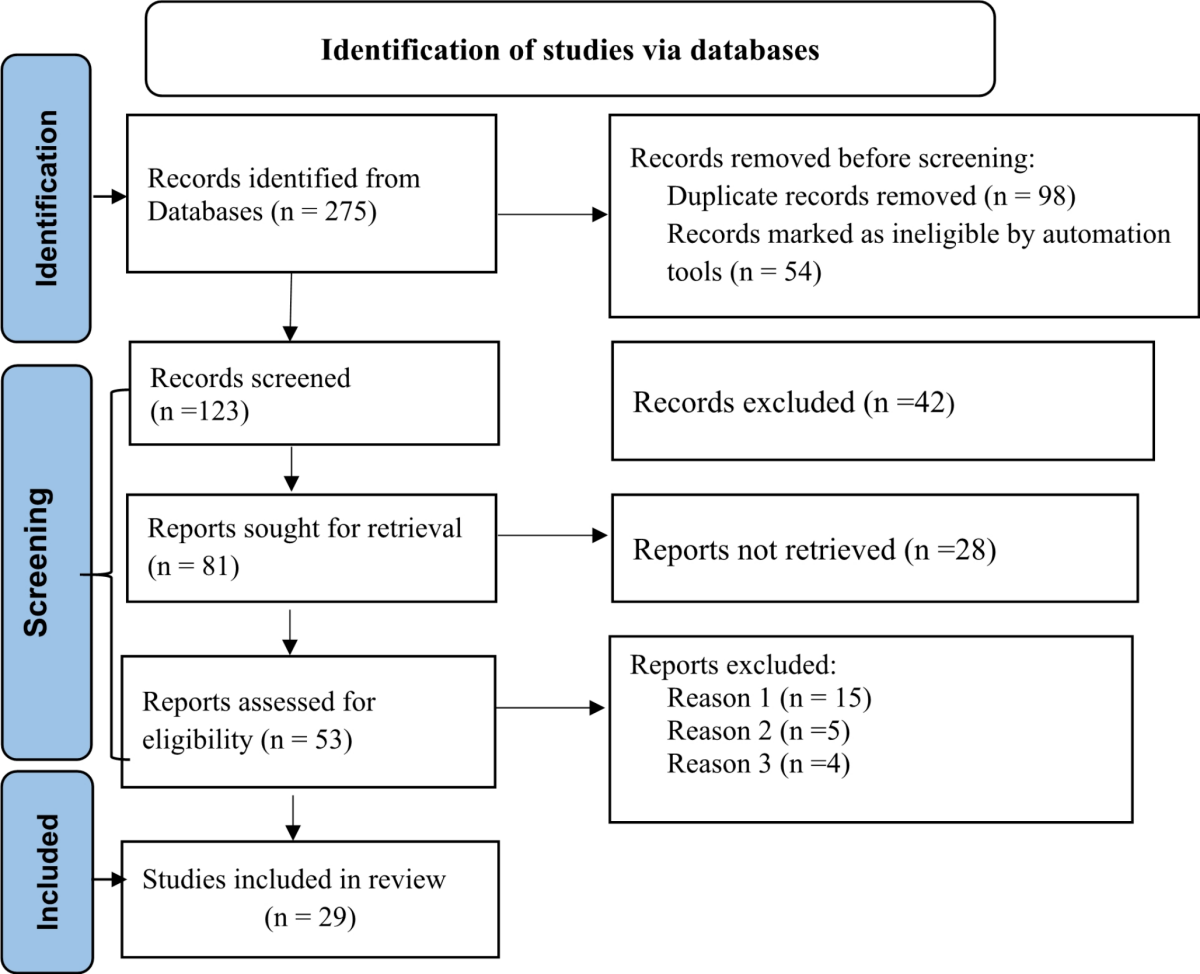
PRISMA flow diagram of the selection of studies, a systematic review of the role of malnutrition and anemia on mortality among Children on ART in sub-Saharan Africa

A comprehensive literature search across multiple databases yielded 275 relevant publications: 101 from PubMed, 86 from Google Scholar, and 78 from other sources. After removing duplicates and screening titles, 152 articles were excluded. Review of 81 abstracts led to the exclusion of 42 more. A full-text assessment of the remaining studies, focusing on predictors of mortality in children under 15, resulted in 27 articles meeting eligibility criteria. Twenty-four studies were excluded due to missing outcomes or data. Finally, 29 studies were included in the systematic review and meta-analysis. The selection process, guided by a PRISMA flow diagram, involved several stages of refinement [32] (Fig. 1).

### Characteristics of the Studies

This systematic review included 27 studies that were conducted across various sub-Saharan African countries between 2010 and the present(2024) and involved a total of 61,796 patients. All studies employed a retrospective cohort design. This review analyzed the impact of severe wasting in 15 studies, underweight in 8 studies and, anemia in 22 studies on HIV mortality. The reviewed studies encompassed a diverse range of sub-Saharan African countries. Ethiopia was the most represented, with 20 studies [10, 13, 20–25, 33–44], two from Kenya [12, 14], and one from South Africa [45]; multicounty studies were also included: one encompassing Malawi, Uganda, and Kenya [46]; another covering Malawi, South Africa, Zambia, and Zimbabwe [47]; and single-country studies conducted in Cote d’Ivoire [48], Tanzania [49], the Democratic Republic of Congo (DRC) [50], Nigeria [51], and Zimbabwe [52] (Table 1).

**Table 1:** Characteristics of the studies included in the review, a systematic review of the role of malnutrition and anemia on mortality among children on ART in sub-saharan Africa

Authors	Study participants	outcome variables assessed	Country	Sample size
Ebissa et al., 2015 [40]	< 5 years	Sever wasting & Anemia	Ethiopia	556
Koye et al., 2012 [43]	< 5 years	Sever wasting	Ethiopia	549
Chekole et al., 2022 [39]	< 15 years	Sever wasting & Anemia	Ethiopia	588
Ebonyi et al., 2014 [19]	< 15 years	Sever wasting	Nigeria	691
Bitew et al., 2017 [24]	< 15 years	Sever wasting & Anemia	Ethiopia	228
Mekonnen et al., 2023 [23]	< 5 years	Sever wasting & Anemia	Ethiopia	415
Alebel et al., 2020 [21]	< 15 years	Sever wasting & Anemia	Ethiopia	553
Tagesse & Abebe, 2020 [44]	< 15 years	Sever wasting	Ethiopia	410
Adem et al., 2014 [33]	< 15 years	Underweight & Anemia	Ethiopia	560
Abrams et al., 2017 [45]	< 5 years	Sever wasting	South African	272
Mulugeta et al., 2017 [10]	< 15 years	Anemia	Ethiopia	757
Auld et al., 2014 [48]	< 15 years	Anemia	Cote d’Ivoire’	2110
Ben-Farhat et al., 2017 [46]	< 15 years	Under weight	Malawi, Uganda and Kenya	3949
Sidamo et al., 2017 [13]	< 15 years	Sever wasting & Anemia	Ethiopia	421
Marie et al., 2022 [22]	< 5 years	Sever wasting	Ethiopia	376
Davies et al., 2014 [47]	< 15 years	Underweight & Anemia	Malawi, South Africa, Zambia & Zimbabwe	12,655
McHugh et al., 2017 [52]	< 15 years	Sever wasting	Zimbabwe	385
Biyazin et al., 2022 [38]	< 15 years	Sever wasting & Anemia	Ethiopia	251
Nyandiko et al., 2022 [12]	< 15 years	Under weight	Kenya	6234
Gebremedhin et al., 2013 [42]	< 15 years	Anemia	Ethiopia	432
Arage et al., 2019 [36]	< 5 years	Sever wasting	Ethiopia	426
Mwiru et al., 2015 [49]	< 15 years	Sever wasting & Anemia	Tanzania	3144
Andargie & Asmleash, 2018 [35]	< 15 years	Anemia	Ethiopia	269
Oumer et al., 2019 [25]	< 15 years	Underweight & Anemia	Ethiopia	243
Molla et al., 2022 [20]	< 15 years	Sever wasting, Underweight & Anemia	Ethiopia	721
Nugent et al., 2014 [50]	< 15 years	Underweight & Anemia	DRC	1010
Alemu et al., 2022 [34]	< 5 years	Anemia	Ethiopia	415

### Quality Assessment and Risk of Bias

Two independent reviewers evaluated the risk of bias in the included studies using the Newcastle‒Ottawa Scale (NOS) for observational studies [53]. The NOS rates research based on three areas selection (up to 4 stars), comparability (up to 2 stars), and outcome (up to 3 stars)—for a total maximum score of 9 stars. High NOS scores indicate stronger methodological quality, with better control for biases such as selection and confounding. In terms of the selection area, the majority of research papers received a rating of 3 out of 4 stars, showing overall satisfactory representativeness and selection methods. Nevertheless, certain studies, such as the one by Ebissa et al. [40], received a lower rating (2 stars) because of possible selection bias, which could impact the representativeness of the study population, indicating potential problems with sample selection or representativeness. Studies with a 3-star rating, such as Koye et al. [43] and Alebel et al. [21], likely had stronger methods for assessing outcomes and, longer tracking periods, whereas some studies [10, 13, 22, 24, 36, 40, 42, 44, 49] were given 2 stars, suggesting varying measurements of outcomes, particularly for anemia. In general, most studies were deemed high quality, receiving a score of 7 stars or higher.

### The Association Between Severe Wasting and HIV-Related Mortality

**Fig. 2:**
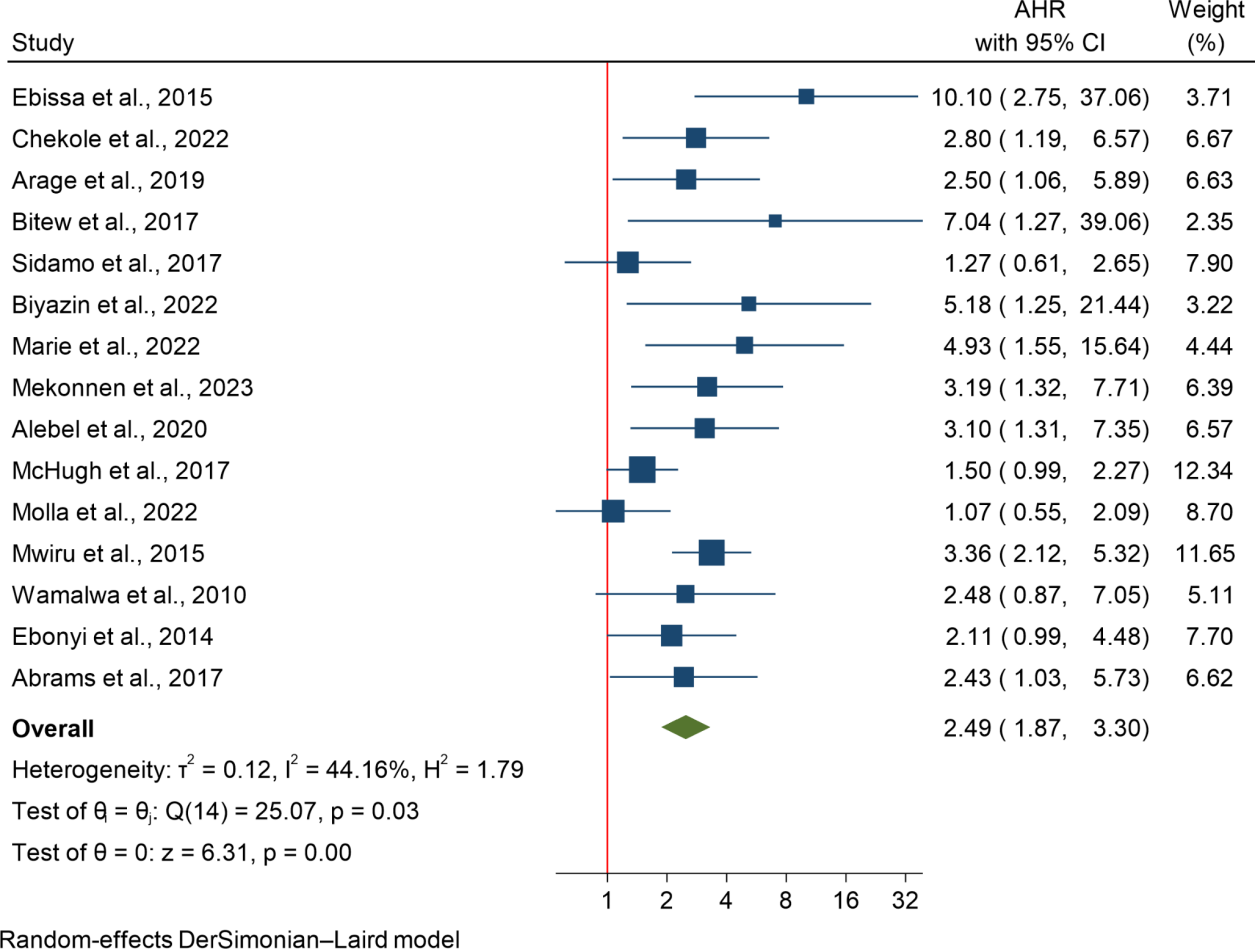
The association between severe wasting and HIV-related mortality, a systematic review of the role of malnutrition and anemia on mortality among Children on ART in sub-Saharan Africa

The impact of severe wasting was assessed in 15 studies involving 9,162 participants. A random-effects meta-analysis was conducted due to moderate heterogeneity (I²=44.16%), showing that severe wasting was associated with more than a twofold increase in the risk of HIV-related death (HR: 2.49; 95% CI: 1.87–3.30). Subgroup analysis by age revealed a higher risk of mortality for children under 5 years (HR: 3.40) compared to those under 15 years (HR: 2.17). Country-based subgroup analysis indicated the greatest effect size in Tanzania (HR: 3.36), followed by Ethiopia (HR: 2.80) and South Africa (HR: 2.43). However, Kenya, Nigeria, and Zimbabwe showed nonsignificant effect sizes. Meta-regression showed that sample size did not significantly influence the pooled estimate (*P* = 0.57), suggesting that heterogeneity was mainly due to age and country differences. Publication bias was assessed through a funnel plot, Begg’s test, and Egger’s test. The funnel plot indicated some studies outside the 95% CI, but with both positive and negative effect sizes. Begg’s and Egger’s tests suggested the presence of publication bias, with p-values < 0.05, indicating small study effects and potential bias (Fig. 2).

### The Association Between Underweight and HIV-Related Mortality

**Fig. 3:**
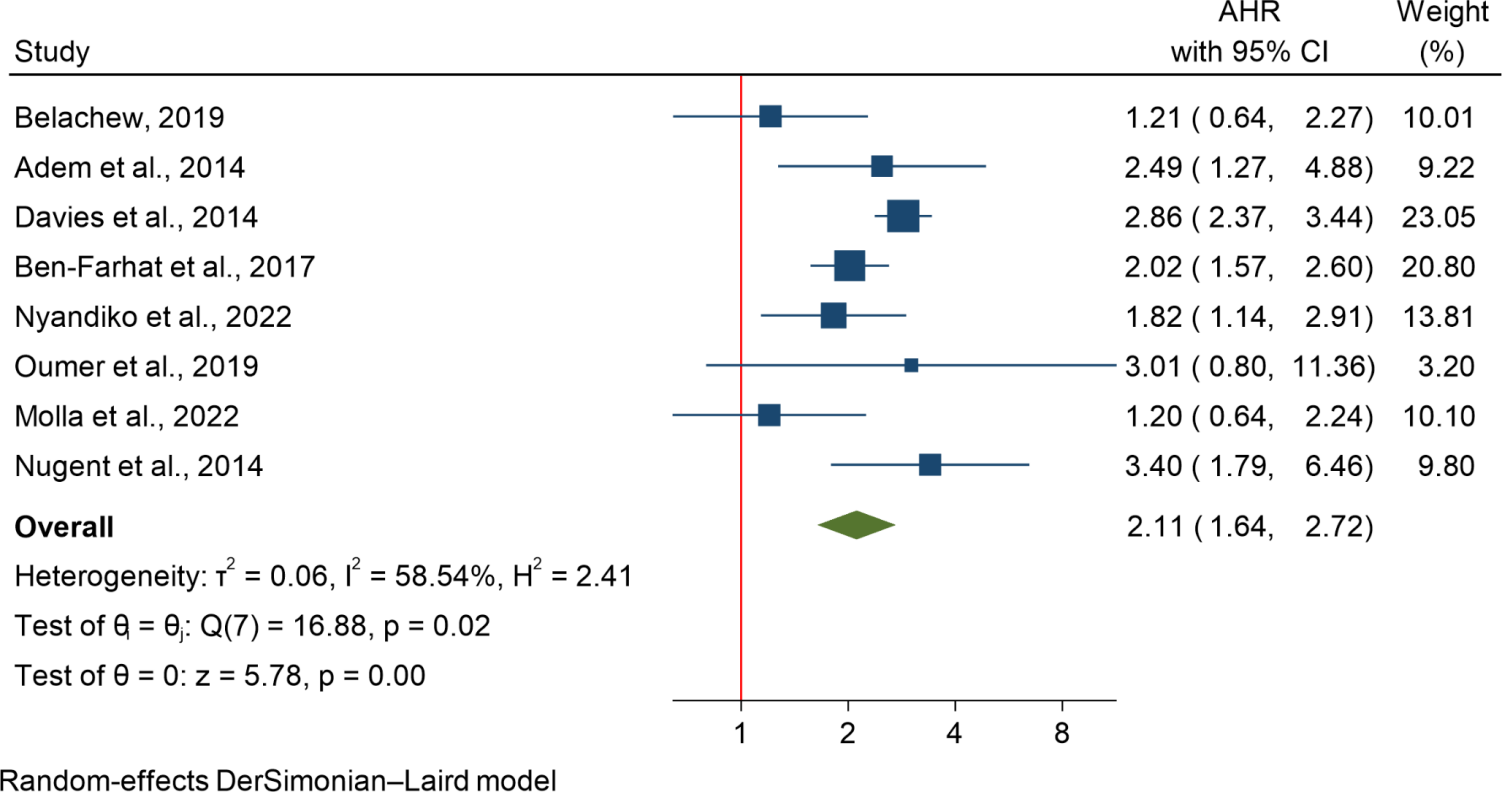
Effect of underweight on HIV-related mortality, a systematic review of the role of malnutrition and anemia on mortality among Children on ART in sub-Saharan Africa

The association between underweight and HIV-related mortality was evaluated in 8 studies [12, 20, 25, 33, 37, 46, 47, 50] involving 1,010 participants. A random-effects meta-analysis was used due to heterogeneity (I²=58.54%). The analysis showed that underweight children had a twofold increased risk of HIV-related mortality (HR: 2.11; 95% CI: 1.64–2.72) (Fig. 3). Subgroup analysis revealed the highest effect in multicounty studies from Malawi, South Africa, Zambia, and Zimbabwe, followed by other regions. Meta-regression showed no significant effect of sample size (*P* = 0.126). Publication bias was not indicated by the funnel plot, Begg’s test (*P* = 0.6398), or Egger’s test (*P* = 0.9015), showing no evidence of small-study effects or bias.

### The Association Between of Anemia and HIV Related Mortality

**Fig. 4:**
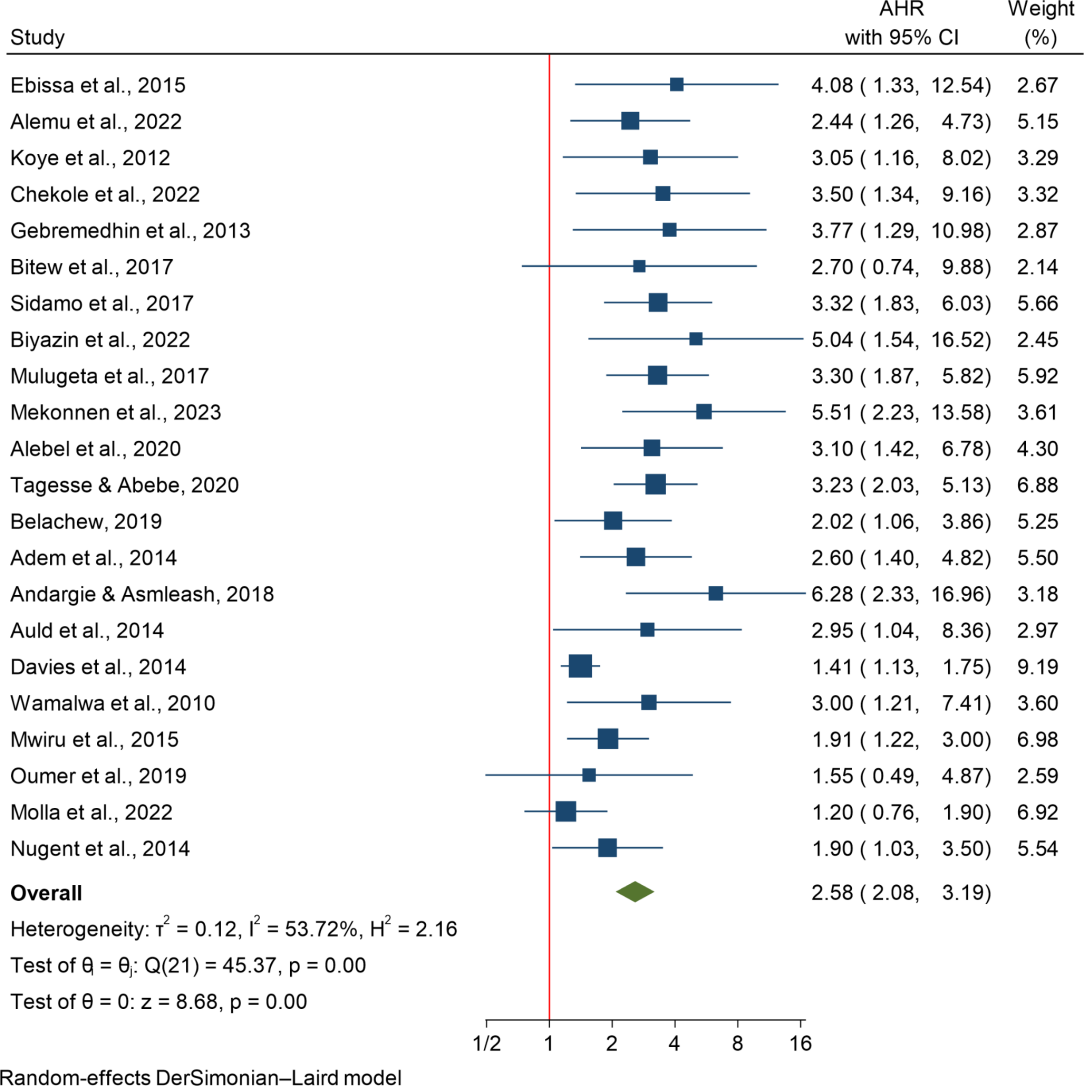
The association between anemia and HIV-related mortality, a systematic review of the role of malnutrition and anemia on mortality among Children on ART in sub-Saharan Africa

Anemia was analyzed in 22 studies involving 26,842 participants using a random-effects model due to significant heterogeneity (I²=53.72%). The analysis revealed that children with anemia had more than twice the risk of mortality (HR: 2.58; 95% CI: 2.08–3.19) (Fig. 4). Subgroup analysis showed a higher risk of death in children under 5 years (HR: 3.31) compared to those under 15 years (HR: 2.45). The largest effect was reported in Kenya (HR: 3.00), followed by Côte d’Ivoire (HR: 2.95) and Ethiopia (HR: 2.83). Meta-regression indicated that smaller sample sizes significantly increased the pooled estimate of mortality risk (*P* = 0.002). Publication bias was assessed through a funnel plot, Begg’s test, and Egger’s test. The funnel plot suggested minimal risk of bias, although a few studies fell outside the 95% CI. Egger’s test indicated publication bias (*P* = 0.0000), while Begg’s test did not show statistical significance (*P* = 0.2363). Since Egger’s test has greater power, it suggests the presence of some publication bias despite Begg’s test showing otherwise.

## Discussion

The review indicated that undernutrition, such as severe wasting and underweight, as well as anemia, was a significant predictor of HIV-related mortality among children. The present analysis revealed a significant association between severe wasting and an increased risk of HIV-related mortality among children on ART in Africa, with a hazard ratio (HR) of 2.49 (95% CI: 1.87–3.30), and underweight, with a twofold increased risk of death (95% CI: 1.64–2.72). These findings underscore the critical impact of malnutrition on the survival of HIV-infected children, especially those under five years old. Several studies corroborate these findings. For instance, a study conducted in India [54] and other countries [55] revealed that severe wasting significantly increased the risk of mortality in HIV-infected children. Similarly, this finding is supported by evidence from East Africa [56], where the pooled prevalence of underweight among HIV-positive children was 41.63%. Malnutrition can be linked to increased HIV-related mortality due to its negative effects on the immune system, treatment effectiveness, and vulnerability to infections. Children who are extremely malnourished and thin typically have a weakened immune system, which hinders their ability to effectively fight viruses and heal from infections [7, 57, 58]. These consistent results highlight the universal impact of malnutrition on the mortality of HIV-infected children, regardless of geographic location. The study’s findings underscore the significant role of malnutrition in increasing HIV-related mortality among children in sub-Saharan Africa, particularly through severe wasting and underweight. Malnutrition compromises the immune system, making HIV-infected children more vulnerable to opportunistic infections and reducing the effectiveness of antiretroviral therapy (ART). This indicates that the challenges of providing malnutrition care among HIV-infected children receiving antiretroviral treatment in Africa are still critical issues, as malnutrition can affect the immunity of the affected children [59]. The context of undernutrition in HIV-infected children is often exacerbated by poverty, food insecurity, and limited healthcare access, all of which make it difficult to provide the necessary nutritional support. Current interventions, such as nutritional rehabilitation and supplementation, integrated with ART, have shown promise in improving survival outcomes [60]. However, challenges in scaling these interventions persist, particularly in rural areas [61]. Further research is required to assess the long-term benefits of integrating nutritional care into routine HIV treatment and to identify the most effective strategies for combating malnutrition in these vulnerable populations.

The current study showed that anemia was a strong indicator of HIV-related death in children, and the risk of death was 2.5 times greater in HIV-infected children with baseline hemoglobin levels less than 10 g/dl than in those with hemoglobin levels greater than or equal to 10 g/dl (HR: 2.52; 95% CI: 2.08–3.19). Studies from multiple centers in Europe have confirmed this discovery [62]. Furthermore, consistent with this report, previous reviews [63, 64] revealed that children with HIV infection who also had anemia had a doubled chance of dying. Anemia is common during the course of HIV infection. This condition further weakens the body’s ability to fight infections, complicating HIV management and worsening health outcomes. In the context of HIV treatment, anemia makes it more difficult for children to recover and respond to ART, increasing the likelihood of poor survival outcomes [29]. It may result directly from being infected with HIV, or it could be caused by nutritional deficiencies, other infections such as systemic fungal and mycobacterium infections, and opportunistic diseases such as lymphoma. Moreover, adverse reactions to antiretrovirals and other drugs can inhibit bone marrow function, causing anemia, thrombocytopenia, and leukopenia [16, 65]. This makes it more difficult for HIV-positive children to survive while receiving antiretroviral therapy (ART). Furthermore, the results show that younger children, especially those under five years old, face a much greater chance of death when suffering from severe malnourishment or anemia. One possible explanation is that younger children have weaker immune systems than older children, making them more vulnerable to the combined impact of HIV, malnutrition, and anemia. The initial five years of life are critical for both physical and cognitive growth [66]. Malnutrition and anemia during this period can have more severe and lasting impacts on a child’s overall health and survival. Therefore, studies should explore the role of micronutrient supplementation and anemia treatment in improving survival outcomes. There is also a pressing need to understand the socioeconomic barriers that prevent malnourished HIV-positive children from receiving comprehensive care. Addressing these gaps will be key to improving the survival and quality of life for children living with HIV in sub-Saharan Africa.

### Limitation of the Study

This review is limited to certain forms of malnutrition, such as severe wasting and undernutrition, while the impact of stunting on HIV-related mortality was not included, potentially reducing the comprehensiveness of the findings. Additionally, since all the included studies were observational, establishing a causal relationship between malnutrition, anemia, and mortality remains challenging.

## Conclusion

This comprehensive analysis, which examined 27 studies in sub-Saharan Africa, offers compelling evidence of the impact of malnutrition and anemia on HIV-related death in children. According to the analysis, severe malnutrition and underweight status had a notable impact on HIV-related death rates. Additionally, anemia was found to be a key factor in predicting HIV-related deaths. Young children under 5 years old were at greater risk of death, showing that malnutrition and anemia during this time could have longer-lasting and more severe consequences on a child’s health and chances of survival. Therefore, the significant link between nutritional status and HIV-related death highlights the importance of combined nutrition and HIV treatment programs in sub-Saharan African countries. Policies should emphasize nutritional support in HIV treatment plans, especially for children under five years of age. Furthermore, health resources need to be used to address both HIV treatment and nutritional support.

## Data Availability

All the data analyzed during this study are included in this published article.
